# Supplementary material for: Dietary quality and cardiometabolic indicators in the USA: A comparison of the Planetary Health Diet Index, Healthy Eating Index-2015, and Dietary Approaches to Stop Hypertension
Source: PLoS One. 2024 Jan 10;19(1):e0296069. doi: 10.1371/journal.pone.0296069 (PMC10781024; doi:10.1371/journal.pone.0296069)
Supplement: S1 Methods — (DOCX) [file pone.0296069.s001.docx]

# S1 Methods.

Smoking data were available from the NHANES Smoking Questionnaire and were coded as a three-level categorical variable [1]: Individuals who reported smoking fewer than 100 cigarettes in their lifetime were considered Never smokers. Individuals who reported no tobacco consumption in the past 30 days were considered Former smokers. Individuals who reported current tobacco use were classified as Current smokers.

Alcohol intake data were available from the NHANES Alcohol Use Questionnaire and were coded as a three-level categorical variable in accordance with Centers for Disease Control and Prevention (CDC) guidelines [2]: Individuals who reported no alcohol consumption in the prior 12 months were classified into None. Men who reported fewer than 14 or fewer drinks per week (average of 2 drinks or fewer per day) and women who reported 7 or fewer drinks per week (average of 1 drink or fewer per day) were classified as Light to Moderate Alcohol Use. Men who report 15 or more drinks per week and women who reported 8 or more drinks per week were classified as having Heavy Alcohol Use.

Physical activity data were available from the NHANES Physical Activity Questionnaire (PAQ). The PAQ asked participants to report how many days in a typical week they participated in the following types of activity: moderate-work, vigorous-work, walk/bicycle, moderate-recreation, and vigorous-recreation. For each type of activity, the questionnaire then probes for how much time was spent doing the respective activity on a typical day. From participants’ responses, we created a binary variable of adequate moderate to vigorous physical activity (MVPA), defined as <2.5 hours (150 minutes) or >=2.5 hours (150 minutes) in line with the recommendations of the CDC [3].

# References

1. Global Adult Tobacco Survey (GATS): Core Questionnaire with Optional Questions, Version 2.0. Atlanta, GA: Centers for Disease Control and Prevention; 2010.

2. Centers for Disease Control and Prevention [Internet]. Excessive Alcohol Use. Atlanta, GA: Centers for Disease Control and Prevention, National Center for Chronic Disease Prevention and Health Promotion;2021 [cited 8 March 2022]. Available from: <https://www.cdc.gov/chronicdisease/resources/publications/factsheets/alcohol.htm>.

3. Centers for Disease Control and Prevention [Internet]. Physical Activity Basics. Atlanta, GA: Centers for Disease Control and Prevention; 2020 [cited 8 March 2022]. Available from: <https://www.cdc.gov/physicalactivity/basics/adults/index.htm>.
