# Supplementary material for: Dietary quality and cardiometabolic indicators in the USA: A comparison of the Planetary Health Diet Index, Healthy Eating Index-2015, and Dietary Approaches to Stop Hypertension
Source: PLoS One. 2024 Jan 10;19(1):e0296069. doi: 10.1371/journal.pone.0296069 (PMC10781024; doi:10.1371/journal.pone.0296069)
Supplement: S1 Table — * Grams per day calculated from dry weight. † To calculate the score for the legumes component, the non-soy and soy subcomponents are each weighted at 0.5. (DOCX) [file pone.0296069.s002.docx]

| S1 Table**:** Scoring criteria for the Planetary Health Diet Index (PHDI)^1^ | | |
| --- | --- | --- |
| **Dietary component** | **Category minimum score** (0 points) | **Category maximum score** (10 points) |
| *Adequacy components* | | |
| Whole grains^*^ | 0 grams | ≥ 75 grams for women  ≥ 90 grams for men |
| Whole fruits (excludes fruit juice) | 0 grams | ≥ 200 grams |
| Non-starchy vegetables | 0 grams | ≥ 300 grams |
| Nuts and seeds | 0 grams | ≥ 50 grams |
| Legumes |  |  |
| Non-soy legumes^*,†^ | 0 grams | 100 grams |
| Soybean/ soy foods^*,†^ | 0 grams | 50 grams |
| Unsaturated oils | 0% of total energy intake | ≥ 10% of total energy intake |
| *Moderation components* | | |
| Starchy vegetables | ≥ 200 grams | ≤ 50 grams |
| Dairy | ≥ 4.08 cup-equivalents | ≤ 1.02 cup-equivalents |
| Red and processed meat | ≥ 300 grams | ≤ 14 grams |
| Poultry | ≥ 58 grams | ≤ 29 grams |
| Eggs | ≥ 120 grams | ≤ 12 grams |
| Fish | ≥ 50 grams | ≤ 15 grams |
| Saturated oils and *trans* fats | ≥ 21% of total energy intake | ≤ 3.5% of total energy intake |
| Added sugar and fruit juice | ≥ 25% of total energy intake | ≤ 5% of total energy intake |
| ^*^ Grams per day calculated from dry weight  ^†^ To calculate the score for the legumes component, the non-soy and soy subcomponents are each weighted at 0.5 | | |
