# Supplementary material for: Dietary quality and cardiometabolic indicators in the USA: A comparison of the Planetary Health Diet Index, Healthy Eating Index-2015, and Dietary Approaches to Stop Hypertension
Source: PLoS One. 2024 Jan 10;19(1):e0296069. doi: 10.1371/journal.pone.0296069 (PMC10781024; doi:10.1371/journal.pone.0296069)
Supplement: S2 Table — * Survey-weighted regression models were adjusted for age, sex, income, education, race/ethnicity, and total energy intake. † mg/dL = milligrams per deciliter. (DOCX) [file pone.0296069.s003.docx]

| S2 Table: Predicted change in continuous and binary cardiometabolic risk factors per one standard-deviation score in Planetary Health Diet Index, Healthy Eating Index-2015, and Dietary Approaches to Stop Hypertension score among undiagnosed participants only, National Health and Nutrition Examination Survey 2003-2018^*^ | | | |
| --- | --- | --- | --- |
|  | PHDI | HEI-2015 | DASH |
|  |  |  |  |
| *Blood pressure* |  |  |  |
| Systolic blood pressure, mm Hg | -0.6 (-1.3, 0.1) | -0.7 (-1.3, -0.1) | -1.0 (-1.5, -0.4) |
| Diastolic blood pressure, mm Hg | -0.1 (-0.6, 0.4) | -0.4 (-1.0, 0.2) | -0.6 (-1.2, -0.1) |
| Predicted probability of high blood pressure | -2.6 (-5.1, -0.1) | -2.3 (-0.3, -4.3) | -2.9 (-5.2, -0.5) |
|  |  |  |  |
| *High-density lipoprotein cholesterol, HDL-C* |  |  |  |
| mg/dL^†^ | 2.1 (1.3, 2.8) | 2.4 (1.8, 3.0) | 1.7 (1.1, 2.3) |
| Predicted probability of low HDL-C | -5.1 (-6.7, -3.4) | -5.5 (-7.0, -4.0) | -3.8 (-5.4, -2.3) |
|  |  |  |  |
| *Fasting plasma glucose, FPG* |  |  |  |
| mg/dL^†^ | -1.2 (-1.8, -0.6) | -1.1 (-1.6, -0.5) | -0.9 (-1.5, -0.3) |
| Predicted probability of high FPG | -3.3 (-6.1, -0.5) | -3.4 (-1.3, -5.5) | -2.7 (-5.0, -0.3) |
|  |  |  |  |
| ^*^ Survey-weighted regression models were adjusted for age, sex, income, education, race/ethnicity, and total energy intake.  ^†^ mg/dL = milligrams per deciliter | | | |
