# Supplementary material for: Dietary quality and cardiometabolic indicators in the USA: A comparison of the Planetary Health Diet Index, Healthy Eating Index-2015, and Dietary Approaches to Stop Hypertension
Source: PLoS One. 2024 Jan 10;19(1):e0296069. doi: 10.1371/journal.pone.0296069 (PMC10781024; doi:10.1371/journal.pone.0296069)
Supplement: S3 Table — * Survey-weighted logistic regression models were adjusted for age, sex, income, education, race/ethnicity, and total energy intake. † * p<0.05, ** p<0.01, *** p<0.001. ‡ Contrast is from Stata’s postestimation margins, dydx command and represents percentage point reduction in predicted probability from Quintile 1 to Quintile 5. (DOCX) [file pone.0296069.s004.docx]

| S3 Table: Predicted probability of cardiometabolic risk factor by quintile of Planetary Health Diet Index, Healthy Eating Index-2015, and Dietary Approaches to Stop Hypertension score, National Health and Nutrition Examination Survey 2003-2018^*,†^ | | | | | |
| --- | --- | --- | --- | --- | --- |
|  | Elevated Waist Circumference | Elevated Blood Pressure | Reduced HDL-C | Elevated Fasting Plasma Glucose | Elevated Fasting Triglycerides |
| *Planetary Health Diet Index* |  |  |  |  |  |
| Quintile 1 | 62.9 (58.8, 67.1) | 45.6 (41.2, 50.0) | 44.8 (40.7, 48.9) | 65.7 (59.9, 71.4) | 24.4 (20.1, 28.7) |
| Quintile 2 | 64.0 (60.0, 68.0) | 46.2 (42.4, 50.0) | 45.4 (40.7, 50.2) | 61.7 (56.4, 67.1) | 24.5 (20.2, 28.8) |
| Quintile 3 | 62.4 (57.9, 67.0) | 46.2 (43.1, 49.2) | 43.9 (40.6, 47.2) | 64.7 (60.0, 69.3) | 23.5 (18.9, 28.1) |
| Quintile 4 | 60.4 (54.9, 65.8) | 42.5 (39.1, 46.0) | 39.2 (34.7, 43.6) | 61.2 (56.2, 66.1) | 17.9 (13.5, 22.2) |
| Quintile 5 | 55.1 (50.7, 59.5) | 38.4 (34.0, 42.8) | 35.2 (32.0, 38.4) | 58.7 (53.8, 63.5) | 21.8 (16.8, 26.8) |
| Contrast, Quintile 1 vs. Quintile 5^‡^ | -7.8^*^ (-14.0, -1.6) | -7.2^*^ (-13.2, -1.3) | -9.6^***^ (-14.6, -4.6) | -7.0 (-14.7, 0.7) | -2.6 (-9.9, 4.7) |
|  |  |  |  |  |  |
| *Healthy Eating Index-2015* |  |  |  |  |  |
| Quintile 1 | 64.8 (60.9, 68.7) | 50.8 (47.3, 54.4) | 47.1 (43.7, 50.6) | 63.8 (60.6, 67.0) | 22.4 (18.0, 26.9) |
| Quintile 2 | 64.1 (60.8, 67.4) | 44.3 (40.2, 48.3) | 46.1 (42.8, 49.4) | 63.6 (58.4, 68.9) | 24.5 (19.8, 29.1) |
| Quintile 3 | 64.1 (59.5, 68.6) | 43.7 (40.4, 47.1) | 42.1 (38.3, 46.0) | 64.5 (59.0, 70.0) | 22.5 (17.6, 27.3) |
| Quintile 4 | 57.6 (51.9, 63.2) | 41.0 (37.8, 44.2) | 36.6 (33.1, 40.1) | 63.9 (58.8, 68.9) | 23.5 (17.7, 29.2) |
| Quintile 5 | 52.9 (47.9, 57.9) | 38.7 (33.6, 43.9) | 35.8 (31.5, 40.2) | 54.5 (48.9, 60.0) | 19.0 (14.0, 24.0) |
| Contrast, Quintile 1 vs. Quintile 5^‡^ | -11.9^***^ (-18.8, -5.0) | -12.1^***^ (-18.8, -5.4) | -11.3^***^ (-15.9, -6.7) | -9.3^**^ (-15.4, -3.2) | -3.5 (-11.1, 4.2) |
|  |  |  |  |  |  |
| *Dietary Approaches to Stop Hypertension* |  |  |  |  |  |
| Quintile 1 | 65.1 (60.9, 69.2) | 49.7 (46.4, 53.1) | 46.1 (42.1, 50.1) | 65.5 (60.9, 70.1) | 22.0 (17.6, 26.3) |
| Quintile 2 | 62.9 (60.1, 65.7) | 46.3 (43.4, 49.3) | 44.0 (40.4, 47.6) | 61.2 (56.2, 66.2) | 25.0 (20.0, 30.0) |
| Quintile 3 | 61.1 (57.1, 65.2) | 44.0 (41.1, 46.9) | 40.1 (35.9, 44.2) | 63.6 (58.8, 68.5) | 21.2 (15.6, 26.7) |
| Quintile 4 | 67.0 (59.9, 74.1) | 39.1 (34.9, 43.3) | 39.9 (33.9, 45.8) | 63.5 (57.9, 69.1) | 27.6 (22.0, 33.3) |
| Quintile 5 | 49.6 (44.8, 54.5) | 39.0 (34.8, 43.3) | 38.2 (33.9, 42.5) | 58.1 (53.0, 63.2) | 17.2 (13.5, 20.9) |
| Contrast, Quintile 1 vs. Quintile 5^‡^ | -15.4^***^ (-21.3, -9.6) | -10.7^***^ (-15.4, -6.0) | -7.9^*^ (-14.2, -1.5) | -7.4^*^ (-13.8, -1.0) | -4.7 (-11.0, 1.5) |
| ^*^ Survey-weighted logistic regression models were adjusted for age, sex, income, education, race/ethnicity, and total energy intake.  ^†^ * p<0.05, ** p<0.01, *** p<0.001  ^‡^ Contrast is from Stata’s postestimation margins, dydx command and represents percentage point reduction in predicted probability from Quintile 1 to Quintile 5 | | | | | |
