# Supplementary material for: Dietary quality and cardiometabolic indicators in the USA: A comparison of the Planetary Health Diet Index, Healthy Eating Index-2015, and Dietary Approaches to Stop Hypertension
Source: PLoS One. 2024 Jan 10;19(1):e0296069. doi: 10.1371/journal.pone.0296069 (PMC10781024; doi:10.1371/journal.pone.0296069)
Supplement: S4 Table — * Survey-weighted logistic regression models were adjusted for age, sex, income, education, race/ethnicity, and total energy intake.† * p<0.05, ** p<0.01, *** p<0.001. ‡ Contrast is from Stata’s postestimation margins, dydx command and represents percentage point reduction in predicted probability from Quintile 1 to Quintile 5. (DOCX) [file pone.0296069.s005.docx]

| S4 Table: Predicted probability of cardiometabolic risk factor by quintile of Planetary Health Diet Index, Healthy Eating Index-2015, and Dietary Approaches to Stop Hypertension value among undiagnosed participants only, National Health and Nutrition Examination Survey 2003-2018^*,†^ | | | |
| --- | --- | --- | --- |
|  | Elevated Blood Pressure  N=4921 | Reduced HDL-C  N=4580 | Elevated Fasting Plasma Glucose  N=3094 |
| *Planetary Health Diet Index* |  |  |  |
| Quintile 1 | 32.7 (28.3, 37.2) | 28.9 (24.5, 33.3) | 63.0 (56.9, 69.1) |
| Quintile 2 | 33.3 (28.5, 38.1) | 30.2 (24.5, 35.9) | 57.7 (51.7, 63.7) |
| Quintile 3 | 31.2 (27.9, 34.5) | 27.8 (23.1, 32.5) | 61.7 (56.6, 66.9) |
| Quintile 4 | 30.5 (25.8, 35.1) | 19.8 (14.8, 24.8) | 56.7 (50.9, 62.4) |
| Quintile 5 | 25.4 (20.5, 30.2) | 17.8 (14.3, 21.3) | 52.9 (47.4, 58.3) |
| Contrast, Quintile 1 vs. Quintile 5^‡^ | -7.3^*^ (-13.8, -0.8) | -11.1^***^ (-15.6, -6.7) | -10.1^*^ (-18.5, -1.7) |
|  |  |  |  |
| *Healthy Eating Index-2015* |  |  |  |
| Quintile 1 | 35.1 (30.8, 39.4) | 30.8 (26.5, 35.0) | 60.5 (56.9, 64.1) |
| Quintile 2 | 31.2 (27.5, 34.9) | 28.5 (23.8, 33.2) | 59.0 (52.9, 65.2) |
| Quintile 3 | 31.0 (27.2, 34.8) | 28.4 (22.8, 34.0) | 61.7 (55.8, 67.5) |
| Quintile 4 | 29.6 (25.4, 33.8) | 19.5 (15.0, 24.0) | 60.3 (54.6, 65.9) |
| Quintile 5 | 26.0 (19.7, 32.3) | 16.4 (12.1, 20.8) | 48.7 (43.4, 54.0) |
| Contrast, Quintile 1 vs. Quintile 5^‡^ | -9.1^*^ (-17.2, -1.0) | -14.3^***^ (-19.0, -9.7) | -11.8^**^ (-18.1, -5.5) |
|  |  |  |  |
| *Dietary Approaches to Stop Hypertension* |  |  |  |
| Quintile 1 | 35.4 (31.0, 39.8) | 29.7 (25.5, 34.0) | 62.3 (57.1, 67.5) |
| Quintile 2 | 33.0 (29.0, 37.0) | 29.3 (24.6, 34.1) | 56.8 (50.6, 62.9) |
| Quintile 3 | 29.8 (26.5, 33.2) | 21.0 (16.9, 25.2) | 60.2 (54.9, 65.6) |
| Quintile 4 | 27.7 (23.3, 32.1) | 26.2 (20.8, 31.6) | 59.9 (53.8, 65.9) |
| Quintile 5 | 27.2 (22.5, 31.9) | 18.1 (13.8, 22.4) | 53.1 (47.5, 58.6) |
| Contrast, Quintile 1 vs. Quintile 5^‡^ | -8.2^*^ (-14.9, -1.6) | -11.6^***^ (-16.7, -6.5) | -9.2^*^ (-16.6, -1.8) |
| ^*^ Survey-weighted logistic regression models were adjusted for age, sex, income, education, race/ethnicity, and total energy intake.  ^†^ * p<0.05, ** p<0.01, *** p<0.001  ^‡^ Contrast is from Stata’s postestimation margins, dydx command and represents percentage point reduction in predicted probability from Quintile 1 to Quintile 5 | | | |
