# Supplementary figures and images for: Dietary quality and cardiometabolic indicators in the USA: A comparison of the Planetary Health Diet Index, Healthy Eating Index-2015, and Dietary Approaches to Stop Hypertension
Source: PLoS One. 2024 Jan 10;19(1):e0296069. doi: 10.1371/journal.pone.0296069 (PMC10781024; doi:10.1371/journal.pone.0296069)

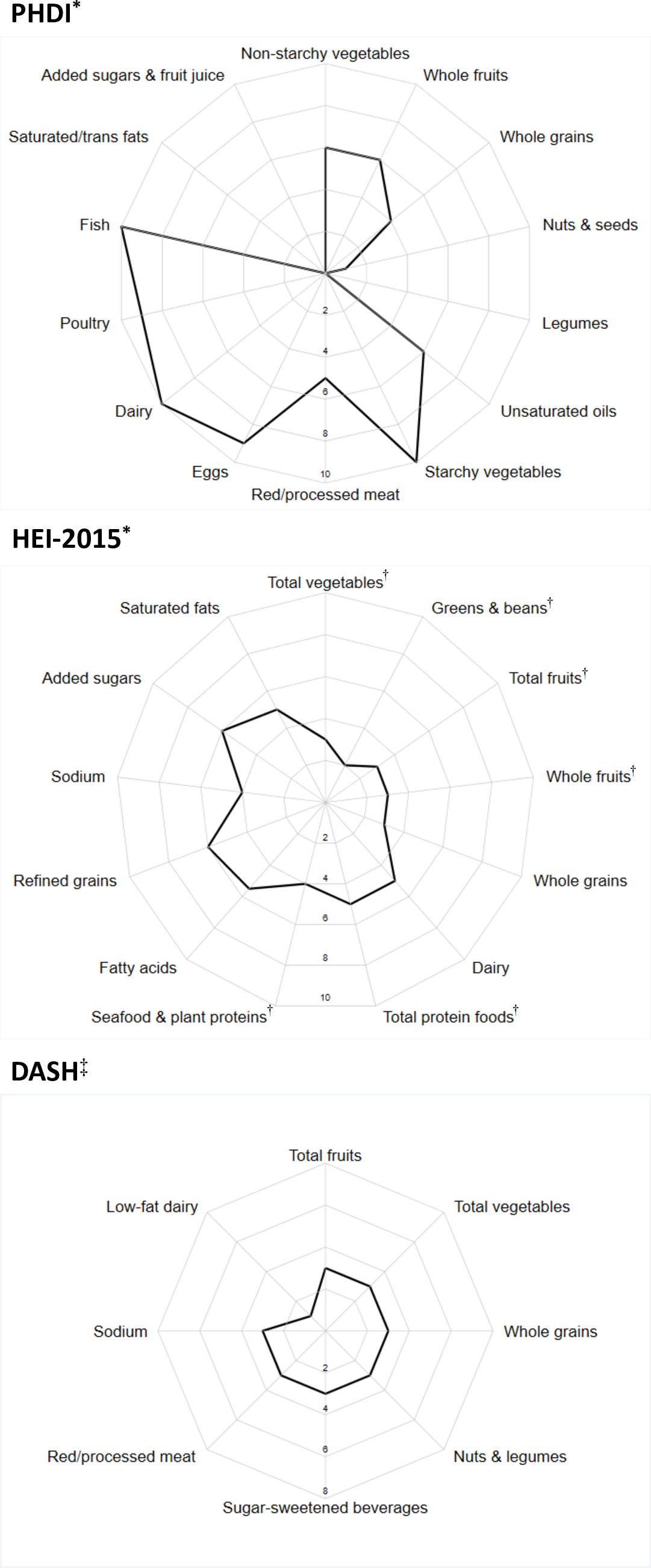

Supplement: S1 Fig — * All dietary pattern component scores range 0–10 unless otherwise noted. † Component score range: 0–5. (TIF) [file pone.0296069.s006.tif]

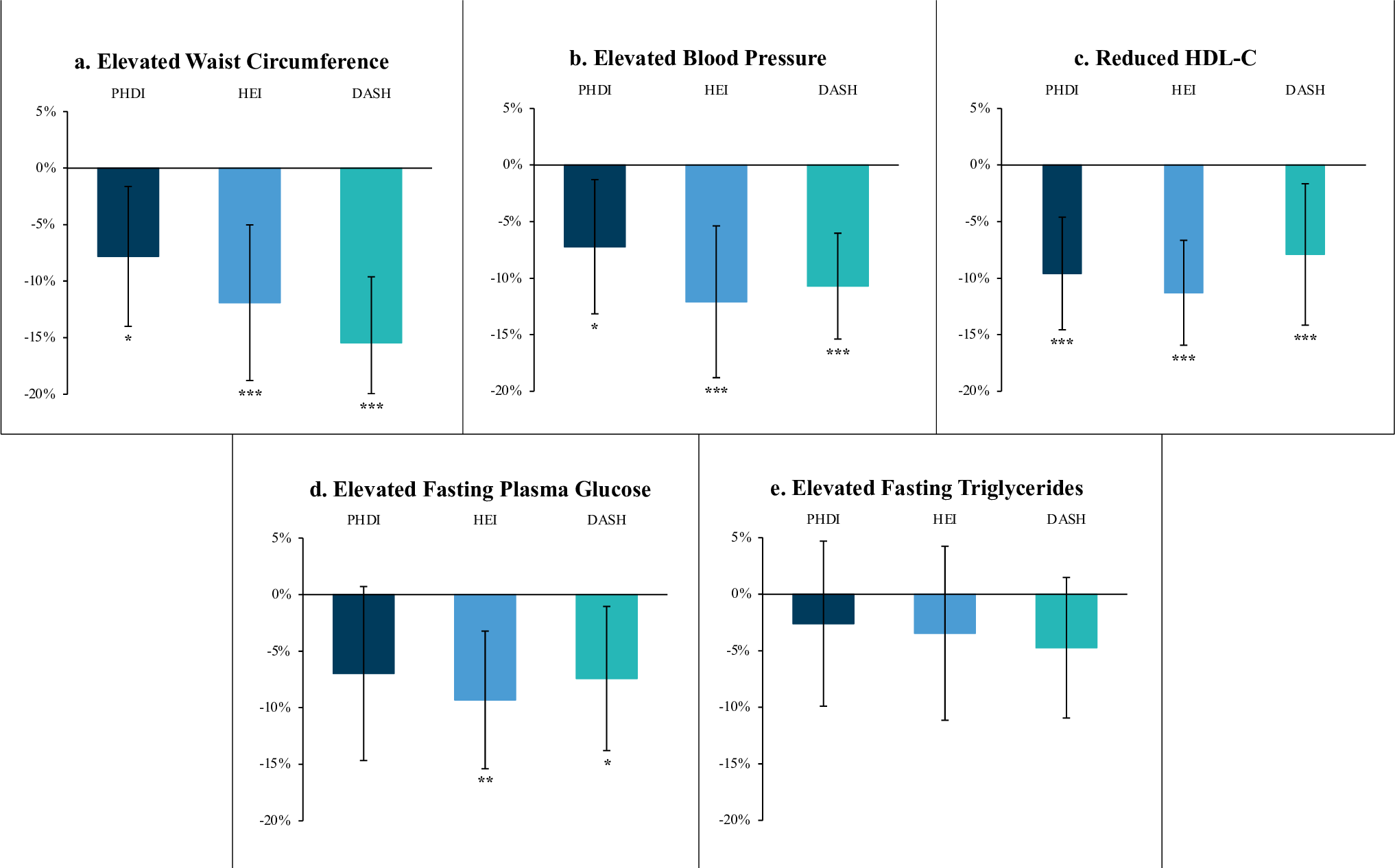

Supplement: S2 Fig — * Logistic regression models were adjusted for age, sex, income, education, and race/ethnicity. * p<0.05, ** p<0.01, *** p<0.001 for the estimated contrast between Quintile 1 and Quintile 5. (TIF) [file pone.0296069.s007.tif]
