# Supplementary material for: Dietary quality and cardiometabolic indicators in the USA: A comparison of the Planetary Health Diet Index, Healthy Eating Index-2015, and Dietary Approaches to Stop Hypertension
Source: PLoS One. 2024 Jan 10;19(1):e0296069. doi: 10.1371/journal.pone.0296069 (PMC10781024; doi:10.1371/journal.pone.0296069)
Supplement: Attachment — Submitted filename: ReviewerResponses.docx [file pone.0296069.s009.docx]

**Journal Requirements:**

When submitting your revision, we need you to address these additional requirements.

1. Please ensure that your manuscript meets PLOS ONE's style requirements, including those for file naming. The PLOS ONE style templates can be found at 
https://journals.plos.org/plosone/s/file?id=wjVg/PLOSOne_formatting_sample_main_body.pdf and 
https://journals.plos.org/plosone/s/file?id=ba62/PLOSOne_formatting_sample_title_authors_affiaffiliat.pdf

**Thank you. We have followed the example formatting above for the manuscript and for the title page.**

2. Please provide additional details regarding participant consent. In the ethics statement in the Methods and online submission information, please ensure that you have specified (1) whether consent was informed and (2) what type you obtained (for instance, written or verbal, and if verbal, how it was documented and witnessed). If your study included minors, state whether you obtained consent from parents or guardians. If the need for consent was waived by the ethics committee, please include this information.

If you are reporting a retrospective study of medical records or archived samples, please ensure that you have discussed whether all data were fully anonymized before you accessed them and/or whether the IRB or ethics committee waived the requirement for informed consent. If patients provided informed written consent to have data from their medical records used in research, please include this information

Once you have amended this/these statement(s) in the Methods section of the manuscript, please add the same text to the “Ethics Statement” field of the submission form (via “Edit Submission”).

For additional information about PLOS ONE ethical requirements for human subjects research, please refer to <http://journals.plos.org/plosone/s/submission-guidelines#loc-human-subjects-research>.

**Thank you. We have updated the language as follows: “The study protocols of the NHANES are approved by the Research Ethics Review Board at the National Center for Health Statistics (NCHS) [14]. This is a retrospective study of data that were fully-anonymized before the authors accessed them. Because the de-identified observational data from the National Health and Nutrition Examination Survey are publicly available for download, this study received a determination of Not Human Subjects Research by the Institutional Review Board at [First Author’s Home Institution].”**

3. Please ensure that you include a title page within your main document.

**Thank you. We have included a title page in the main document and formatted according to the guidelines available at the link provided by the Journal.**

4. We note that you have included the phrase “data not shown” in your manuscript. Unfortunately, this does not meet our data sharing requirements. PLOS does not permit references to inaccessible data. We require that authors provide all relevant data within the paper, Supporting Information files, or in an acceptable, public repository. Please add a citation to support this phrase or upload the data that corresponds with these findings to a stable repository (such as Figshare or Dryad) and provide and URLs, DOIs, or accession numbers that may be used to access these data. Or, if the data are not a core part of the research being presented in your study, we ask that you remove the phrase that refers to these data.

**Thank you for this feedback. The cited results are not a core part of the research being presented in the study and the phrase “data not shown” has been removed.**

5. Please include captions for your Supporting Information files at the end of your manuscript, and update any in-text citations to match accordingly. Please see our Supporting Information guidelines for more information: http://journals.plos.org/plosone/s/supporting-information.

**Thank you. We have included the captions for our Supporting Information files at the end of the manuscript. We have additionally uploaded each Supporting Information file individually per the guidelines on the PLOS ONE website.**

Reviewer #1: Comments to authors:

The main objective of this study was to explore the association between PHDI, HEI, and DASH with cardiometabolic biomarkers. The authors revealed that the association between HEI and DASH with the majority of cardiometabolic biomarkers was stronger than the association between PHDI with cardiometabolic biomarkers.

1. Another very interesting topic that has been addressed by dear authors but not deeply focused on has been socio-economic disparities. Other researchers have shown that the more people deviate from a healthy eating pattern, the more cardiometabolic biomarkers worsen both in people with risk factors such as diabetes and in healthy people. This has been well-established in many studies on food insecurity. In many communities where people are food insecure, their cardiometabolic biomarkers have worsened. In very simple language, food insecurity means insufficiency and imbalance in receiving all food groups in the daily food pattern.

**Thank you for this comment. We wholeheartedly agree that food insecurity contributes to dietary disparities and to poorer cardiometabolic outcomes, particularly for historically disadvantaged populations. An analysis of food insecurity was beyond the scope of the present manuscript. We did find disparities in dietary quality for all three indices (PHDI, HEI-2015, and DASH) by key sociodemographic indicators, including income, education, and race/ethnicity, and reported on these in the results section. In the discussion we talk about the disparities in dietary quality that were observed in our study and that are consistent with dietary disparities that have been documented repeatedly in the literature (lines 331-332). We also mention that these disparities are due to a variety of structural factors that make accessibility and affordability of health food difficult, and that climate change has the potential to exacerbate these disparities (lines 332-339). We then mention that policy solutions are needed to address upstream determinants of health disparities and improve dietary quality (lines 339-341).**

**In this way, we hope that our study contributes to the important conversation about disparities in dietary quality, particularly for historically disadvantaged populations, and adds to the calls for policy solutions to ensure equitable access to healthy diets for all Americans.**

2. Authors should know another point well that all parts of a manuscript/article must follow a certain homogeneity and respond to the main objectives of the study. Therefore, the authors are strongly requested to do the same in the discussion of the manuscript as they showed the correlation difference between the three indicators with selected biomarkers in the results of the study.

**Thank you for this comment. We believe that the discussion does follow a standard manuscript format, as we first summarize our results, then qualitatively compare the indices (which aligns with our descriptive analyses), summarize and discuss the implications of the similar correlations with cardiometabolic (which aligns with our primary objective and main regression analyses), discuss SD (which aligns with our secondary objective), and then move on to strengths and limitations. We have added some language to the discussion to signal which part of the results we are referring to in a given paragraph (e.g. “our descriptive analyses”, “our primary analyses”).**

**Please note that because of the similarities between the indices for our primary results, and because of the sheer quantity of results, we did not use discussion space to go through each biomarker individually. We believe such an approach would have been redundant for a reader and distracting from the other points we raised in the discussion.**

3. Another point that the authors know very well is that more than 26 years have been studied on the healthy eating index and more than 20 years on the DASH model, and these two patterns have well proven their role in the nutritional health of the people of a society. Therefore, avoid one-sidedness in the discussion which is the most essential part of the article.

**Thank you for your comment. We agree with the reviewer that HEI and especially DASH are valuable public health nutrition tools. We spend more time discussing the PHDI since it is a novel index and may not be familiar to readers as HEI or DASH, whose benefits are already well-established.**

**In the revision, we present a more balanced discussion of the three indices in several ways:**

- **First, in the second and third paragraphs that compare PHDI, HEI-2015, and DASH scoring, we have emphasized that we are comparing the PHDI to two well-established ways of measuring healthy diets (lines 284-285). We have also reworded to emphasize “differences” between the dietary indices (lines 295-296) rather than making comparisons of “worse quality” as in the previous version.**
- **Second, we have taken out the sentence “Despite these differences, overall healthy plant-based diets – such as the PHDI - have been associated with lower cardiometabolic risk in a large US-based cohort study [40], suggesting that improved long-term adherence to the PHDI would similarly be associated with decreased cardiometabolic risk over time.”** **We replaced this sentence with “Despite these differences, all three diets have healthy plant-based options, which have not only been associated with lower cardiometabolic risk in a large US-based cohort study, but also have significant benefits for environmental sustainability [40].” (lines 326-328).**
- **Finally, in the last paragraph of the discussion, we mention that ideal policy solutions “would address upstream determinants of health disparities and would lead to improvements in dietary quality measured by PHDI, HEI-2015, and DASH (lines 339-341).”**

4. The researchers who initially designed, implemented, and presented the healthy eating index and DASH aimed to implement the policies to promote access to healthy diets for vulnerable populations in the US.

**Thank you for this comment. We agree that both HEI and DASH could be leveraged to reduce dietary disparities, and acknowledge in both our Introduction section (lines 75-75) and Discussion section (lines 331-332) that there are well-documented disparities for both of these indices. We further mention that policies could lead to improvements in both HEI and DASH as well as in PHDI (lines 339-341).**

5. The strengths and limitations section should be presented in a separate section under the subtitle of “the strengths and limitations” before the conclusion.

**We appreciate this suggestion. We have delimited “Limitations and strengths” as a subsection under the Results and discussion main heading. Please note that Results and discussion is now one heading rather than two per journal formatting guidelines (available from https://journals.plos.org/plosone/s/file?id=wjVg/PLOSOne_formatting_sample_**

**main_body.pdf).**

6. The last point is that the conclusion at the end of the article should be modified according to the obtained results.

**Thank you. We agree with the reviewer’s comment. We have modified the conclusion to first focus on the primary results of the study (i.e., the similar correlations with cardiometabolic indicators) at a high-level, and then have a high-level summary of the need for policy solutions. The conclusion now reads: “Our analysis suggests that sustainability-focused dietary recommendations, which we operationalized using the PHDI, have similar benefits for cardiometabolic risk factors as HEI-2015 and DASH. There is a need for effective policy solutions to support healthy diets overall, and particularly for populations suffering from a high burden of diet-related disease. Including sustainability in dietary guidelines can have environmental co-benefits while promoting population-level cardiometabolic health.” (lines 359-364).**

Reviewer #2: I would thank the authors for this valuable article. I have some questions:

1- In Table 1, please say why you report the three indices (PHDI, HEI, and DASH) with median (IQR) instead of Mean (SD).

**Thank you for this comment. We have updated the table to report the mean (SD) of the scores rather than the median (IQR).**

2- In Table 5, You did not report any p-values for linear and logistic regressions. Please add p-values to test whether, for example, high waist circumference risk significantly differs in the three indices.

**Thank you for this comment. We have added columns for the pairwise test of the beta coefficients from each regression model into table 5. We have also added asterisks indicating the difference from zero for each predicted difference provided in table 5, and updated the table legend accordingly.**
