# Supplementary material for: Dietary quality and cardiometabolic indicators in the USA: A comparison of the Planetary Health Diet Index, Healthy Eating Index-2015, and Dietary Approaches to Stop Hypertension
Source: PLoS One. 2024 Jan 10;19(1):e0296069. doi: 10.1371/journal.pone.0296069 (PMC10781024; doi:10.1371/journal.pone.0296069)
Supplement: Attachment — Submitted filename: ReviewerResponses.docx [file pone.0296069.s010.docx]

**Journal comments:**

Please apply the suggestions of the respected reviewer to present the p-values for the logistic regression statistical test in cardiometabolic biomarkers between the three dietary guidelines. The consent of the statistical expert reviewer of your manuscript will be the condition of acceptance of your article.

**Statistical expert reviewer comments:**

With comment 2 ("In Table 5, You did not report any p-values for linear and logistic regressions. Please add p-values to test whether, for example, high waist circumference risk significantly differs in the three indices.") I want you to perform a test to compare three indices simultaneously not pair-wise comparisons.

Notice: I have a statistician's point of view on your results. If it is not necessary to compare these three indices (PHDI, HEI, and DASH) from a nutritional point of view, please do not change Table 5

**We thank the reviewer and the journal for their comments and the opportunity to revise the submission. We have now jointly compared the PHDI, HEI-2015, and DASH indices using a Wald test and report a single p-value. These were done for the linear and logistic regression models whose results are reported in Table 5.**
